# Longitudinal chemokine profile expression in a blood-brain barrier model from Alzheimer transgenic versus wild-type mice

**DOI:** 10.1186/s12974-018-1220-7

**Published:** 2018-06-13

**Authors:** J. Vérité, T. Janet, D. Chassaing, B. Fauconneau, H. Rabeony, G. Page

**Affiliations:** 1 EA3808, molecular Targets and Therapeutics of Alzheimer’s disease, University of Poitiers, 86073 Poitiers, France; 2 SATT Grand Centre- Société d’Accélération du Transfert de Technologie, 8, rue Pablo Picasso, 63000 Clermont-Ferrand, France

**Keywords:** Alzheimer, Blood-brain barrier, Mouse, Primary cell culture, Chemokines, Peripheral blood mononuclear cells, Longitudinal study

## Abstract

**Background:**

Alzheimer’s disease is widely described since the discovery of histopathological lesions in Mrs. Auguste Deter in 1906. However to date, there is no effective treatment to deal with the many cellular and molecular alterations. The complexity is even higher with the growing evidence of involvement of the peripheral blood mononuclear cells (PBMCs). Indeed, monocytes and T cells are shown in the cerebral parenchyma of AD patients, and these cells grafted to the periphery are able to go through the blood-brain barrier (BBB) in transgenic mouse models. It is known that BBB is disrupted at a late stage of AD. Chemokines represent major regulators of the transmigration of PBMCs, but many data were obtained on AD animal models. No data are available on the role of AD BBB in a healthy brain parenchyma. Therefore, the purpose of this study was to analyze the longitudinal chemokine profile expression in a BBB model from AD transgenic mice versus wild-type (WT) mice.

**Methods:**

A primary mouse BBB model was used with a luminal compartment either AD or WT and an abluminal compartment WT consisting of astrocytes and microglia. PBMCs were extracted by a ficoll gradient and incubated in the transwell with a direct contact with the luminal side, including the endothelial cells and pericytes. Then, the complete BBB model was incubated during 48 h, before supernatants and cell lysates were collected. Chemokines were quantified by X-MAP® luminex technology.

**Results:**

Abluminal CX3CL1 production increased in 12-month-old AD BBB while CX3CL1 levels decreased in luminal lysates. CCL3 in luminal compartment increased with aging and was significantly different compared to AD BBB at 12 months. In addition, abluminal CCL2 in 12-month-old AD BBB greatly decreased compared to levels in WT BBB. On the contrary, no modification was observed for CCL4, CCL5, and CXCL10.

**Conclusion:**

These first findings highlighted the impact of AD luminal compartment on chemokine signature in a healthy brain parenchyma, suggesting new therapeutic or diagnostic approaches.

**Electronic supplementary material:**

The online version of this article (10.1186/s12974-018-1220-7) contains supplementary material, which is available to authorized users.

## Background

Alzheimer’s disease is a neurodegenerative disorder with well-described brain lesions associated with multiple cellular and molecular alterations, some of which have been extensively studied but with no satisfactory results in terms of diagnostic tools or therapeutic targets [1–6]. Although protected by the blood-brain barrier (BBB), the brain maintains close, dynamic communication with peripheral blood [7–10]. However, this neurovascular unit (NVU) is very vulnerable to various stresses including the amyloid peptide (Aβ) and its inflammatory environment that it generates [11–14]. Indeed, numerous in vitro and in vivo studies demonstrated the Aβ-endothelium interactions, leading to many structural and functional changes at the BBB [14, 15]. Furthermore, Aβ increased adherence and transmigration of monocytes across the BBB in vitro [16, 17] and in vivo studies through CCL2/CCR2 axis [18–20]. Besides, T cells as mononuclear cells also accumulate in AD brain [21–23]. The role of these peripheral cells in parenchyma is debated, either favoring the increased microglia activation, Aβ deposition, impaired cognitive functions, secreting pro-inflammatory cytokines or playing a defense role to senescent microglia [24, 25]. For this last role, it was shown that monocytes would be more effective than resident microglia which expresses a negative genetic variant of Triggering Receptor Expressed on Myeloid Cells 2 (TREM2) associated to a decreased plaque-associated microgliosis [26, 27]. In addition, regulatory T cells play a beneficial role in AD models during the early phase of the disease, by slowing disease progression and modulating microglial responses to Aβ deposition [28].

It should be noted that most data from the literature on the BBB failure and the presence of PBMCs in parenchyma are observed very late on postmortem samples. On the contrary, recent imaging studies to assess the neurovascular dysfunction in AD could be used to early detect abnormalities of the BBB permeability [29]. At the level of the BBB, the passage of PBMCs must be finely regulated according to the stage of the disease. Among regulators, many studies indicate not only the role of chemokines in this transmigration of PBMCs through BBB but also their role in driving crosstalk between neurons, glial cells, and peripheral immune cells [30–32]. In AD, microglia, astrocytes produce many chemokines and participate to neuroinflammation of AD [33–36]. Many data also showed that T cells and monocytes of AD patients overexpressed some chemokines (CCL2, CCL4, CXCL8, CXCR2) compared to age-matched controls, priming their transendothelial migration [37, 38]. Several studies using genetic tools modulating chemotaxis in different murine models of AD by down- or over-expressing some chemokines (CCR5, CCL5, CCL2, CCR2, CX3CR1) showed a direct impact on amyloid burden and memory decline [39–41]. Recently, monocyte-derived macrophages (MDM) infiltration was found in very aged transgenic mice. MDM highly expressed activation markers at basal state. In contrast, microglia exhibited an activated phenotype only with normal aging and Aβ pathology. At the later stages of the pathology, chemokines (CCL2, CCL3, CCL4, CXCL1) were mostly expressed in GFAP-positive astrocytes and were detected in few Iba-1-positive microglia and NeuN-positive neurons [42].

All these findings underlined the role of PBMCs in particular monocytes and T cells in the pathophysiology of AD and also the role of chemokines at the level of BBB. However, the chemokine environment was always studied in AD animal models or AD patients versus controls. Any data is available about the role of AD BBB in healthy brain parenchyma. Therefore, the purpose of this study was to analyze the longitudinal chemokine profile expression in a BBB model from AD transgenic mice versus WT mice. This primary mouse BBB model was prepared with a luminal compartment either AD or WT and an abluminal compartment WT consisting of astrocytes and microglia. PBMCs were incubated in the transwell with a direct contact with the luminal side, including endothelial cells and pericytes. The main results showed that abluminal CX3CL1 production increased in 12-month-old AD BBB while CX3CL1 levels decreased in luminal lysates. CCL3 in luminal medium in 12-month-old WT BBB increased and significantly different compared to 12-month-old AD BBB. In addition, CCL2 in abluminal medium of 12-month-old AD BBB greatly decreased compared to levels in WT BBB. The expression of the CCL4, CCL5, and CXCL10 chemokines was not modified. These first results obtained in an integrated BBB model including main cells of the NVU highlighted some chemokines as potential therapeutic targets and other as future biomarkers in AD.

## Methods

### Chemical products

Ficoll Histopaque®-1077, newborn calf serum (NBCS), phytohaemagglutin (PHA), dimethylsulfoxide (DMSO), sodium fluoride (NaF), phenylmethylsulfonyl fluoride (PMSF), triton X-100, Fluorescein isothiocyanate-Dextran of 4-kDa (FD4), Rhodamine 123, Zosuquidar hydrochloride or LY-335979, protease and phosphatase inhibitor cocktails, and all reagent-grade chemicals for buffers were obtained from Sigma (Saint-Quentin Fallavier, France). The tissue digestion kit and myelin isolation beads were purchased from Miltenyi Biotech (Paris France). RPMI 1640 medium, DMEM, 5000 units of penicillin (base) and 5000 units of streptomycin (base)/mL mixture (PS), gentamycin, glutamine, trypsine-EDTA (0.05%), newborn calf serum, Quant-it protein assay from Gibco-Invitrogen (Fisher Scientific, Illkirch, France), EndoGRO-LS Complete Culture Media Kit, X-MAP® luminex Kit for cytokine/chemokine assay from Merck-Millipore (Saint Quentin Yvelines, France).

### The mouse blood-brain barrier model

In this study, a mouse model of BBB was developed in the laboratory. The methodology followed was to extract endothelial cells, pericytes from the brain using commercially available kits for tissue digestion and elimination of myelin (Miltenyi Biotech, Paris France). The cells are cultured separately in respective media: EndoGRO-LS Complete Culture Media Kit for endothelial cells and DMEM completed with 20% of newborn calf serum, 1% of PS, and 0.1% of gentamycin for pericytes. At day 10, the BBB is mounted on 12-well inserts (Polyester (PET) Membrane Transwell-Clear Inserts from Corning (Fisher Scientific distributor, Illkirch, France) and ready for experiments 3 days later. The luminal compartment included endothelial cells and pericytes seeded on the transwell and PBMCs suspended in the culture medium. All these primary cells were isolated either from AD or WT mice. The abluminal compartment represented the brain parenchyma corresponded to primary astrocytes/microglia co-cultures extracted from the brain of WT mice and prepared as described previously [43, 44]. In the study, there were two different configurations of the BBB model: the luminal part (the endothelial cells, pericytes, and PBMCs) was prepared from either AD mouse and the BBB was named “AD BBB” or WT mouse and the BBB was named “WT BBB” throughout the manuscript. The abluminal part always consisted of cells from WT mice (Additional file 1).

In this model, PHA-stimulated mouse PBMCs were added in the transwell insert, in direct contact with the luminal side. Finally, the complete BBB model was incubated during 48 h at 37 °C in 95% humidified 5% CO_2_ cell culture incubator, before supernatants and cell lysates were collected.

### Animals

Male hemizygote B6C3-Tg (APPswe, PS1dE9)85Dbo (Stock # 004462) and female wild-type mice (B6C3F1, Stock # 10010) were purchased from Jackson Laboratories (Bar Harbor, Maine USA) and bred to create colonies of APPswePS1dE9 and wild-type (WT) mice. As described in the website of Jackson laboratory, two expression plasmids (Mo/HuAPP695swe and PS1-dE9) were designed to each be controlled by independent mouse prion protein (PrP) promoter elements, directing transgene expression predominantly to central nervous system (CNS) neurons. The Mo/HuAPP695swe transgene expresses a “humanized” mouse amyloid beta (A4) precursor protein gene modified at three amino acids to reflect the human residues and further modified to contain the K595N/M596L mutations linked to familial Alzheimer’s disease (FAD). The PS1dE9 transgene expresses a mutant human presenilin 1 carrying the exon-9-deleted variant (PSEN1dE9) associated with FAD. In this mouse model of AD, occasional amyloid deposits can be found as soon as 6 months of age and plaques are abundant in the hippocampus and cortex at 9 months. Furthermore, it also shows amyloid plaques and its associated inflammatory response at early stage of the mouse life, and it progressively increases with age [45, 46]. An agreement was obtained from the High Council of Biotechnology for transgenic animals in 2011 and renewed in 2015 (agreement number: 2040). All animal care and experimental procedures conformed with the French Decree number 2013-118, 1 February 2013 NOR: AGRG1231951D in accordance with the European Community guidelines (directive 2010/63/UE). In France, any sampling of biological material in animals for ex vivo experimentation does not require prior agreement of the ethics committee (COMOTHEA Poitou Charentes). These experiments in the animal are classified outside the scope of the French Decree number 2013-118. All efforts were made to minimize animal suffering as well as the number of animals used. The animals were housed in a conventional state under adequate temperature (23 ± 3 °C) and relative humidity (55 ± 5%) control with a 12/12 h reversed light/dark with access to food and water ad libitum.

### Extraction of peripheral blood monocellular cells

Blood collection from deep anesthetized mice with 80 mg/kg IP pentobarbital (MSD, Santé Animale, Beaucouze, France) was performed by cardiac puncture and transferred into 10 mL BD Vacutainer® tube. Then, PBMCs were isolated using a Ficoll® Histopaque-1077 density gradient and cultured as previously described for human PBMCs [47–49]. PBMCs collected from blood samples of four mice were seeded in 500 μL of complete culture medium (RPMI 1640 medium completed with 10% of newborn calf serum, 1% of PS, and 20 μg/mL PHA) in 12-well plates during 24 h and then transferred into the upper side of transwell insert of the BBB model for 48 h. The most common agents used to stimulate PBMCs are either PHA, lipopolysaccharide (LPS), or β-amyloid peptide (Aβ). Here, we chose the PHA as a mitogen to assess chemokine levels in different conditions as described in our previous studies [48–50] and in those of other authors [51–53]. LPS is capable of altering the proliferative response of lymphocytes by priming of monocytes [54], and the beta amyloid peptide shows a resistance to the proliferation of PBMC from AD patients compared to controls [55].

### Blood-brain barrier permeability

Paracellular permeability was assessed by using a fluorescent molecule tracer (4-kDa Fluorescein Isothiocyanate-Dextran, FD4) as a valuable indicator of barrier integrity. The purpose of this technique is to evaluate the BBB tightness prepared from AD and WT mice at 3, 6, and 12 months of age in our experimental conditions. The flux of FD4 (16 mg/mL) diluted in Hank’s Balanced Salt Solution (HBSS: 0.4 g/L KCl, 0.06 g/L KH_2_PO_4_, 8 g/L NaCl, 0.35 g/L NaHCO_3_, 0.048 g/L Na_2_HPO_4_, 1 g/L D-Glucose, 0.14 g/L CaCl_2_, 0.1 g/L MgCl_2_, 6H_2_O, 0.1 g/L MgSO_4_, 7H_2_O) through cellular layers was determined after taking medium samples (50 μL) from the upper and lower chambers at incubation time by 0 and 60 min, and each sample were transferred into Nunc FluoroNunc/LumiNunc 96-Well plates. Measurements of fluorescence were performed by using a Varioskan Flash® microplate reader (Fisher ThermoScientific, Illkirch, France) with excitation at 485 nm and emission at 515 nm. All experiments were performed using 12 mm Corning® Transwell inserts with pore size of 0.4 μm inserted into Corning® 12-well plates. Two conditions were used during this assay: permeability was evaluated with insert without cells, i.e., only pre-coated insert (control) and in BBB model without PBMCs. Results are expressed as a permeability coefficient (cm s^− 1^). Permeability values in the order of magnitude of 10^− 6^ cm/s are considered good values in scientific literature [56].

### Trans-endothelial electrical resistance measurements

Trans-endothelial electrical resistance (TEER) across the luminal side on transwells was determined using a Millicell® ERS-2 (Electrical Resistance System) device (Millipore, Molsheim, France) with a STX01 electrode. Then, the measured tissue resistance of cells grown on transwell filter inserts was corrected by substracting the blank resistance evaluated across an empty pre-coated transwell insert (without cells), and multiplied by the effective surface area (1.12 cm^2^), to give TEER in Ohms × cm^2^ (Ω cm^2^).

### P-glycoprotein activity

P-gp activity was assessed by measuring the efflux of Rhodamine 123 (a P-gp probe) on cells grown in 12-well plates. Before each experiment, culture cell medium was removed and replaced by fresh culture media containing 2 μM Rhodamine 123 added in the luminal chamber for 1 h at 37 °C. Uptake of 50 μL samples from the upper and lower chambers after 0 and 60 min were performed, and samples were transferred into Nunc FluoroNunc/LumiNunc 96-Well plates. All experiments were performed using Corning® Transwell inserts with 12 mm diameter and pore size of 0.4 μm inserted into Corning®12-well plates. Two conditions were tested during this assay: the efflux of rhodamine 123 was evaluated with insert without cells, i.e., only pre-coated insert (control) and in a BBB model without PBMCs. Measurements of fluorescence were performed by using a Varioskan Flash® microplate reader (Fisher ThermoScientific, Illrkich, France) with excitation at 500 nm and emission at 524 nm. The results represent the percentage of Rhodamine in the abluminal compartment compared to the control BBB (without cell) which is fixed at 100% rhodamine accumulation in abluminal medium.

### X-MAP® Luminex assay

After 48 h in the presence of PBMCs, cells were lysed in 150 μL of lysis buffer (50 mM Trizma® base, 50 mM NaCl pH 6.8, extemporaneously supplemented with 1% Triton X100, 1 mM PMSF, 50 mM NaF, 1% protease inhibitor cocktail, and 1% phosphatase inhibitor cocktail). The lysates were then sonicated (output control 2, duty cycle 20%, 5 pulsations) and centrifuged at 15000*g* for 15 min at 4 °C. The supernatants were saved and analyzed for protein determination using Quant-it protein assay with Qubit® material. Samples were frozen at − 80 °C until X-MAP® luminex assay.

Cells in luminal compartment were designed “luminal lysates” and those in abluminal compartment “abluminal lysates.”

Mouse Cytokine/Chemokine Luminex custom 5-plex kits (for CCL2, CCL3, CCL4, CCL5, and CXCL10) and 1-plex kit (for CX3CL1) were purchased from Millipore (Saint Quentin Yvelines, France). The assay was performed in 96-well plates. All reagents, standards from a range of concentrations (3.2 to 25,000 pg/mL) and quality controls were prepared according to the Millipore instructions. The plates were incubated on a plate shaker at 800 rpm for overnight at 4 °C in a darkroom. Assessment was made using luminex-200® instrument and xPONENT® software. Fifty beads/assays were collected, and median fluorescence intensities (MFIs) were measured. Sensitivity limit was 6.7, 7.7, 11.9, 2.7, 0.8, and 8 pg/mL for CCL2, CCL3, CCL4, CCL5, CXCL10, and CXC3CL1, respectively. Expression of each analyte has been measured in culture media (medium 1 for the luminal compartment and medium 2 for the abluminal compartment) and cellular lysates named “luminal lysates” and “abluminal lysates.” MFIs were converted to concentrations (pg/mL) using the equation of standard range of the appropriate chemokine using Milliplex® Analyst Software. Results were expressed as picograms per milligram of protein for cellular lysates and picograms per milliliter for media.

### Statistical analysis

Results were analyzed by using GraphPad Prism® software. For all data, we performed the D’Agostino-Pearson normality test that has guided us to the choice of non-parametric tests because the sample size *n* was too small and that the probability of normality test was greater than 0.05. Comparisons between two groups, non-matched pairs, were accomplished by using non parametric Mann-Whitney test. For more than two groups, non-parametric Kruskall-Wallis test followed by Dunn’s test were performed. The level of significance was *P* < 0.05.

## Results

### Assessment of the barrier tightness and functionality

**Fig. 1:**
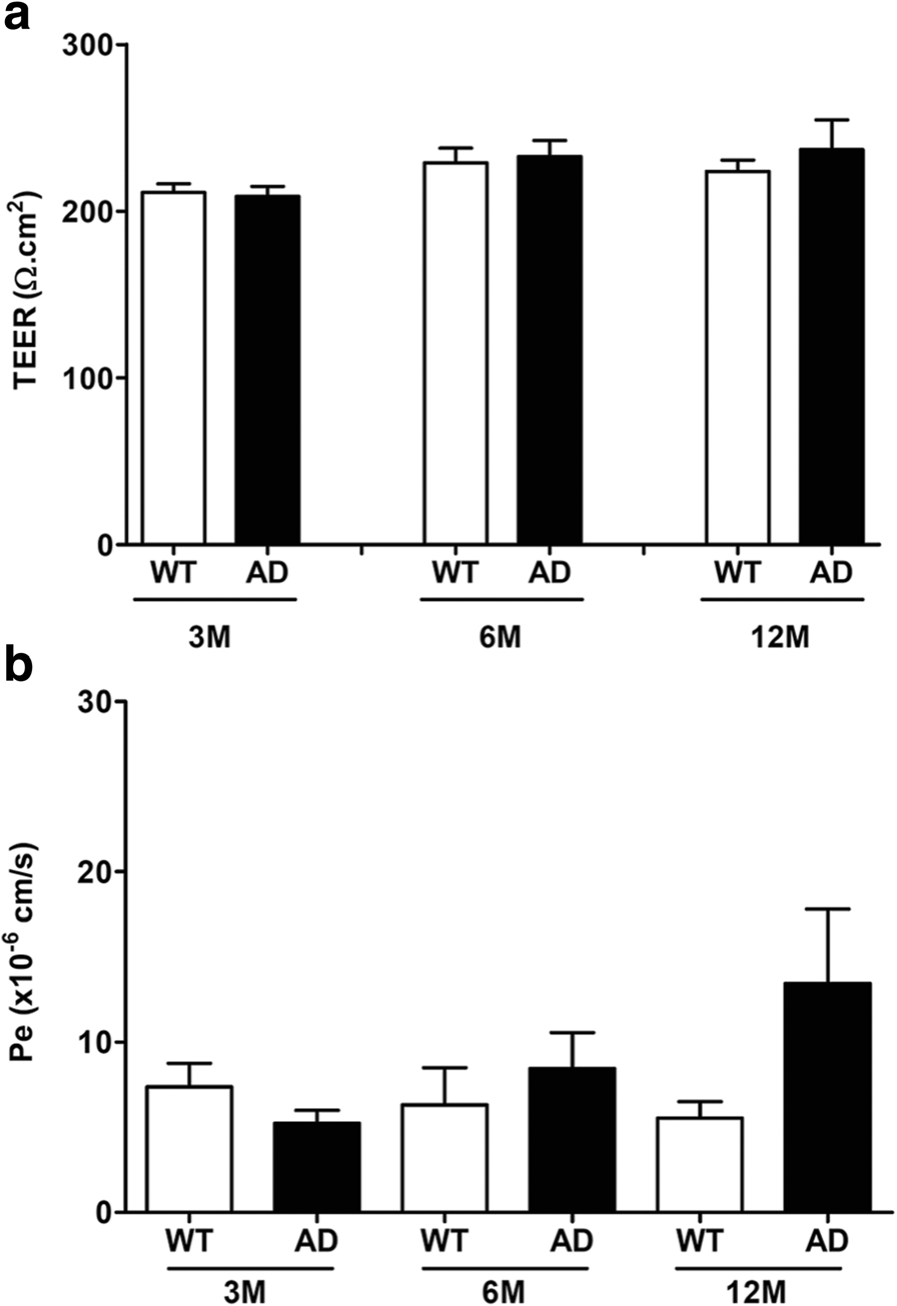
**a** TEER measurements in the murine BBB models at the ages of 3 months (*n* = 6 for WT, *n* = 4 for AD), 6 months (*n* = 7 for WT, *n* = 9 for AD), and 12 months (*n* = 14 for WT, *n* = 6 for AD). Results are expressed in Ω.cm^2^. **b** Permeability values (Pe) for Fluorescein Isothiocyanate-Dextran FD4 in the murine BBB models at the ages of 3 months (*n* = 7 for WT, *n* = 6 for AD), 6 months (*n* = 8 for WT, *n* = 7 for AD), and 12 months (*n* = 12 for WT, *n* = 5 for AD). Results are expressed in cm s^− 1^

In addition to electrical resistance TEER, the paracellular transport capacity has been determined by measuring permeability of FD4 as a molecular tracer. However, no difference was observed between WT and AD BBB models whatever the age of mice in terms of TEER (Fig. 1a) and the paracellular transport capacity of FD4 (Fig. 1b).

**Fig. 2:**
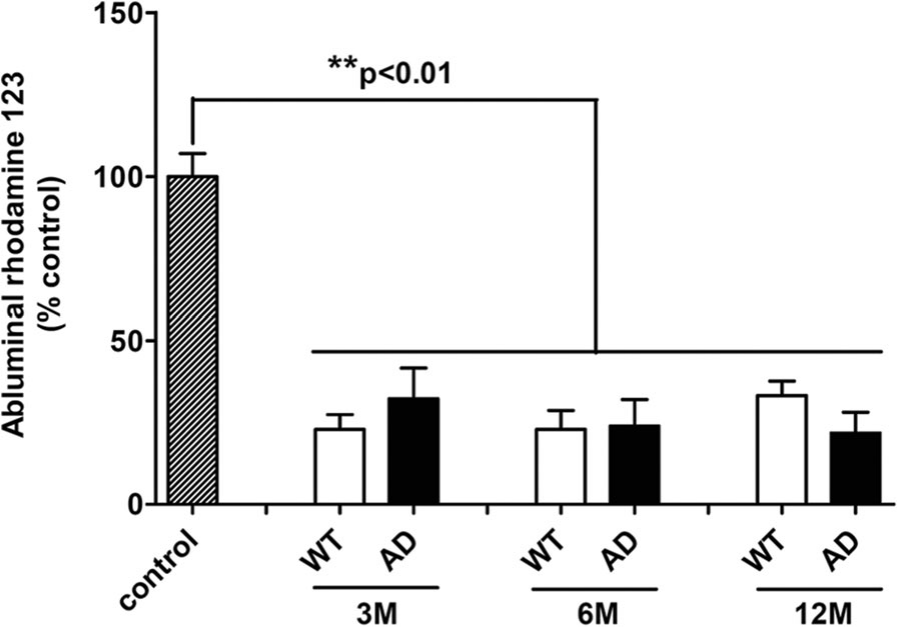
P-glycoprotein activity assay by measuring efflux of Rhodamine 123 (a P-gp probe) in BBB models at the ages of 3 months (*n* = 7 for WT and AD), 6 months (*n* = 4 for WT and AD), and 12 months (*n* = 8 for WT, *n* = 4 for AD). Abluminal fluorescence intensities of rhodamine 123 are expressed in percentage of control (*n* = 17) corresponding to a coated transwell without cells as indicated in methods

Furthermore, to determine the functionality of BBB, rhodamine 123 was used as a P-gp probe due to the important role of P-gp for the transport barrier function of the BBB [57, 58]. In our experimental conditions, results showed BBB functionality with a significant reduction of the abluminal rhodamine 123 rates by about 74% in all groups compared to control insert without cells (Fig. 2). However, the rates of rhodamine 123 in abluminal compartment were no different between WT and AD BBB models whatever the age of mice (Fig. 2).

In order to check the tightness of the BBB after 48 h of PBMC incubation, we measured TEER only because the measurement of paracellular permeability with Dextran-FITC could interfere with the chemokine assay using the X-MAP® luminex technology with magnetic beads grafted with antibodies specifically directed against antigens. In Table 1 are grouped averages of TEER calculated and expressed in Ω cm^2^. We found that these values were comparable to those obtained before the addition of PBMCs regardless of the age and phenotype of the mice used to extract the cells that make up the BBB model.

**Table 1:** TEER in the BBB after 48 h of incubation with PBMCs

	WT 3M	AD 3M	WT 6M	AD 6M	WT 12M	AD 12M
TEER	225.70 ± 9.52	218.40 ± 5.60	233.20 ± 15.80	224.30 ± 5.89	208.50 ± 5.08	244.20 ± 19.60

TEER was measured in the murine BBB models at 3 (*n* = 4), 6 (*n* = 8), and 12 months (*n* = 8)

Results are expressed as mean ± SEM in Ω cm^2^

### Chemokine expression in BBB models

In our experimental conditions, no significant difference was observed in CCL4, CCL5, and CXCL10 expression levels in each group regardless of age and between the two groups at 3, 6, and 12 months (Table 2).

**Table 2:** Longitudinal chemokine levels in all fractions of BBB models

Chemokines	BBB fractions	3M		6M		12M	
		WT	AD	WT	AD	WT	AD
CX3CL1	M1	83.46 ± 20.27	74.53 ± 38.46	139.50 ± 31.67	99.73 ± 23.25	99.76 ± 17.93	70.38 ± 10.29
	Abluminal lysates	0.98 ± 0.54	1.175 ± 0.45	1.13 ± 0.42	1.01 ± 0.45	0.79 ± 0.27	1.03 ± 0.19
CCL2	M1	20,371 ± 9295	13,182 ± 4167	9458 ± 2368	8076 ± 2567	12,963 ± 3943	4383 ± 1520
	PB lysates	1039 ± 307	577.60 ± 284.30	221.40 ± 59.11	264.10 ± 102.50	1007 ± 547.80	120.70 ± 31.74
	Luminal lysates	531.10 ± 227.70	335.10 ± 126.20	214.50 ± 54.57	480.30 ± 226.80	270 ± 76.21	114.40 ± 25.77
	Abluminal lysates	393.60 ± 142	278.50 ± 172.10	139 ± 40.94	269.20 ± 126.40	249.90 ± 137.9	53.42 ± 10.36
CCL3	M2	79.96 ± 25.51	33.02 ± 6.81	311.50 ± 126.40	231.70 ± 86.12	717.40 ± 271.30	179.90 ± 110
	PB lysates	2.33 ± 2.18	7.93 ± 1.89	5.24 ± 1.78	2.77 ± 1.34	14.19 ± 6.34	4.48 ± 2.03
	Luminal lysates	14.55 ± 4.93	11.62 ± 2.59	14.68 ± 3.02	12.09 ± 3.10	28.71 ± 7.63	7.83 ± 2.30
	Abluminal lysates	7.82 ± 3.43	0.31 ± 0.16	5.24 ± 1.31	2.89 ± 1.34	8.06 ± 2.67	3.13 ± 1.53
CCL4	M1	375 ± 125.90	333.50 ± 83.34	599.40 ± 186.90	675.50 ± 231	1135 ± 354.20	241.40 ± 110.30
	M2	332 ± 117.40	167.80 ± 41.30	624.50 ± 254.20	880.90 ± 325.20	1503 ± 463.10	342.30 ± 226.90
	PB lysates	6.98 ± 3.55	5.99 ± 2.22	11.99 ± 5.28	10.51 ± 3.60	18.95 ± 7.31	5.76 ± 3.91
	Luminal lysates	11.15 ± 5.06	8.34 ± 3.39	11.76 ± 4.87	13.78 ± 4.89	15.95 ± 5.52	9.60 ± 3.35
	Abluminal lysates	12.31 ± 3.28	2.46 ± 2.31	20.17 ± 7.61	13.98 ± 5.17	267.60 ± 117.20	4.40 ± 2.60
CCL5	M1	16.96 ± 7.98	14.84 ± 4.86	81.18 ± 40.05	62.77 ± 25.69	128.30 ± 63.82	13.07 ± 5.30
	M2	15.02 ± 6.90	6.25 ± 3.13	82.91 ± 42.46	73.45 ± 38.29	138.60 ± 72.71	7.65 ± 1.99
	PB lysates	0.81 ± 0.34	1.51 ± 0.32	2.98 ± 1.54	2.32 ± 0.81	7.77 ± 4.07	2.56 ± 1.58
	Luminal lysates	2.43 ± 0.93	2.31 ± 0.43	6.58 ± 3.37	3.71 ± 1.35	5.46 ± 2.07	2.82 ± 1.00
	Abluminal lysates	1.28 ± 0.62	0.34 ± 0.19	2.34 ± 1.06	1.55 ± 0.68	2.83 ± 1.40	1.19 ± 0.77
CXCL10	M1	526.30 ± 189.80	283.60 ± 81.98	304.50 ± 98.88	261.10 ± 74.48	318.40 ± 61.75	246.40 ± 79.64
	M2	325.10 ± 109.50	189.30 ± 54.77	426.50 ± 203.50	505.10 ± 202	438.30 ± 177.6	185.10 ± 42.96
	PB lysates	21.98 ± 11.41	20.06 ± 12.06	49.71 ± 24.08	46.80 ± 17.02	38 ± 20.53	60.77 ± 27.67
	Luminal lysates	22.87 ± 14.16	18.91 ± 5.84	35.84 ± 13.50	46.72 ± 14.66	46.18 ± 17.28	17.19 ± 7.94
	Abluminal lysates	13.54 ± 9.27	8.03 ± 4.23	20.75 ± 8.35	23.58 ± 9.54	28.95 ± 15.76	13.91 ± 9.18

Chemokine levels (CX3CL1, CCL2, CCL3, CCL4, CCL5, and CXCL10) in all fractions of BBB models at 3 (WT *n* = 4–5, AD *n* = 5–6), 6 (WT *n* = 10–11, AD *n* = 8–9), and 12 months (WT *n* = 8–11, AD *n* = 4–6) were analyzed by the Luminex® xMAP® assay containing a mixture of beads specific for each chemokine as described in the “Methods” section. Chemokine levels are mean ± SEM and expressed in picograms per milliliter for luminal and abluminal media and in picograms per milligram of protein for cell lysates. Statistical analyses revealed no significant difference

#### CX3CL1 levels

**Fig. 3:**
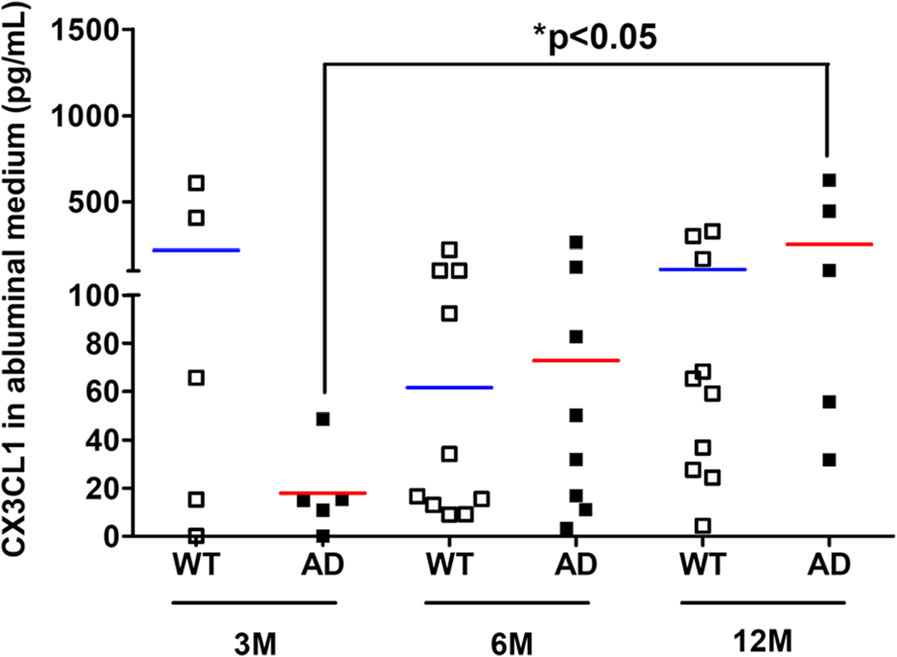
CX3CL1 expression in abluminal media (M2) from WT and AD BBB models at the ages of 3 months (*n* = 5 for WT and AD), 6 months (*n* = 10 for WT, *n* = 8 for AD), and 12 months (*n* = 10 for WT, *n* = 5 for AD). CX3CL1 levels were analyzed by the 1-plex Luminex® xMAP® assay as described in the “Methods” section and expressed in picograms per millilitre. The mean is represented by a colored line following the origin of cell types (blue for WT and red for AD cells, respectively). ^*^P < 0.05 in AD BBB model at 12 months compared to AD BBB models at 3 months by a Kruskal-Wallis test with a Dunn’s multiple comparison test

**Fig. 4:**
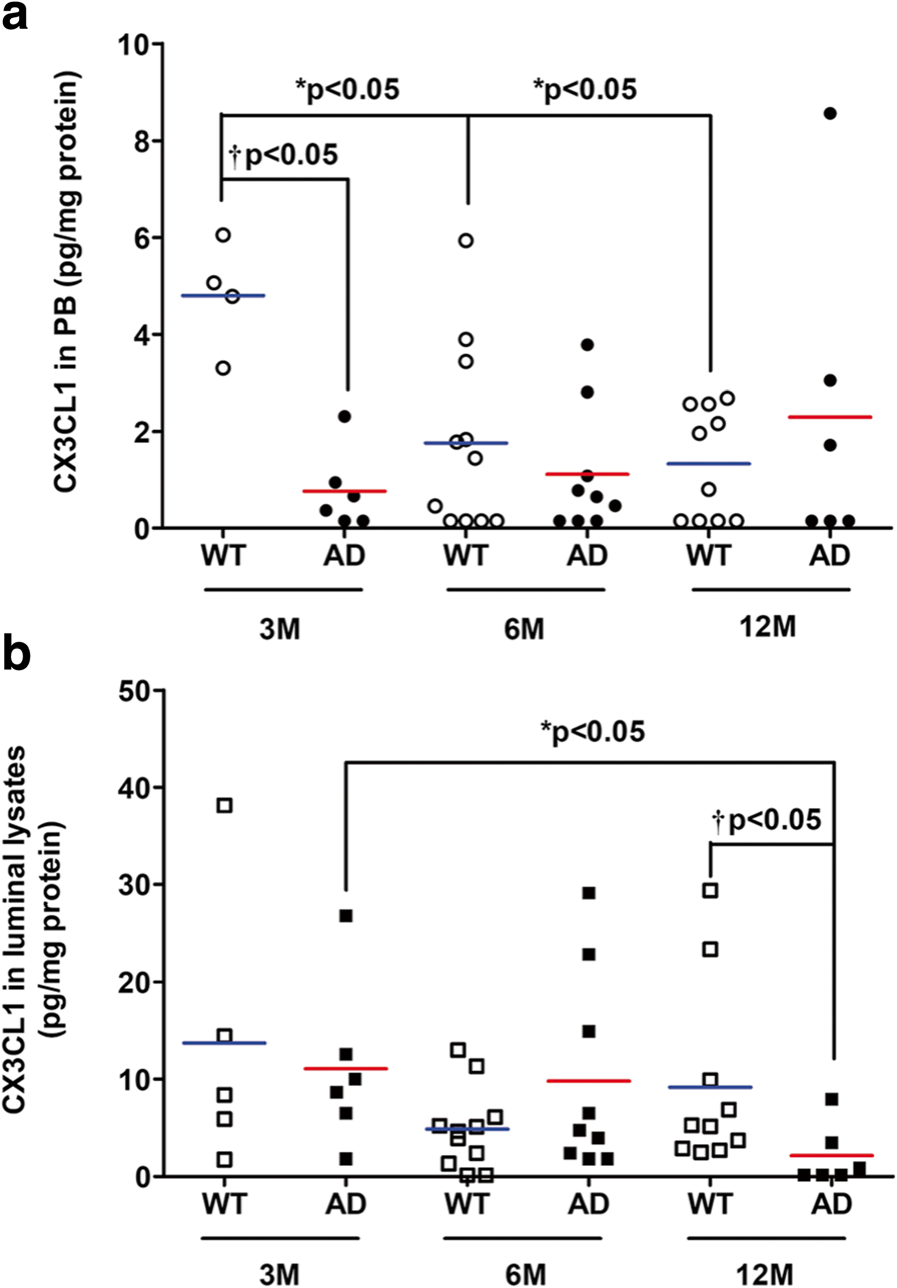
**a** CX3CL1 expression in PBMCs from WT and AD BBB models at the ages of 3 months (*n* = 4 for WT, *n* = 6 for AD), 6 months (*n* = 11 for WT, *n* = 9 for AD), and 12 months (*n* = 10 for WT, *n* = 6 for AD). **b** CX3CL1 expression in luminal lysates from WT and AD BBB models at the ages of 3 months (*n* = 5 for WT, *n* = 6 for AD), 6 months (*n* = 11 for WT, *n* = 9 for AD), and 12 months (*n* = 10 for WT, *n* = 6 for AD). CX3CL1 were analyzed by the 1-plex Luminex® xMAP® assay as described in the “Methods” section. Chemokine levels are expressed in picograms per milligram of protein. The mean is represented by a colored line following origin of cell types (blue for WT and red for AD cells, respectively). **a** ^*^P < 0.05 in PBMCs from WT BBB models at 6 and 12 months compared to WT BBB model at 3 months by Kruskal-Wallis test with a Dunn’s multiple comparison test. ^†^*P* < 0.05 in PBMCs from AD BBB models compared PBMCs from WT BBB model at 3 months by Mann-Whitney’s test. **b** ^*^P < 0.05 in luminal lysates from AD BBB models at 12 months compared to AD BBB models at 3 months by Kruskal-Wallis test with a Dunn’s multiple comparison test. ^†^*P* < 0.05 in luminal lysates from AD BBB model compared to WT BBB model at 12 months by Mann-Whitney’s test

Results showed that CX3CL1 levels were highly increased by 14.06-fold in the abluminal media (M2) of a 12-month-old AD BBB model compared to 3-month-old AD BBB model (Fig. 3). No difference was found in the luminal media (M1) and in abluminal lysates between WT and AD BBB models or with different age groups (Table 2). However, results showed a 2.72-fold decrease of CX3CL1 levels in PBMCs from WT BBB model at 6 versus 3 months, and a 3.59-fold decrease in PBMCs from WT BBB model at 12 versus 3 months (Fig. 4a). No modification with age was found in PBMCs from AD BBB models. However, CX3CL1 levels in PBMCs from AD BBB model were decreased by 6.28-fold compared to PBMCs from WT BBB models at 3 months (Fig. 4a). In luminal lysates, a CX3CL1 decrease by 78% was observed in 12-month-old AD BBB model compared to 3-month-old AD BBB (Fig. 4b). Moreover, the CX3CL1 expression in luminal lysates of AD BBB model at 12 months was 4.35-fold lower than that in WT BBB model at the same age (Fig. 4b). No difference was noted in the abluminal lysates (Table 2).

#### CCL2 levels

**Fig. 5:**
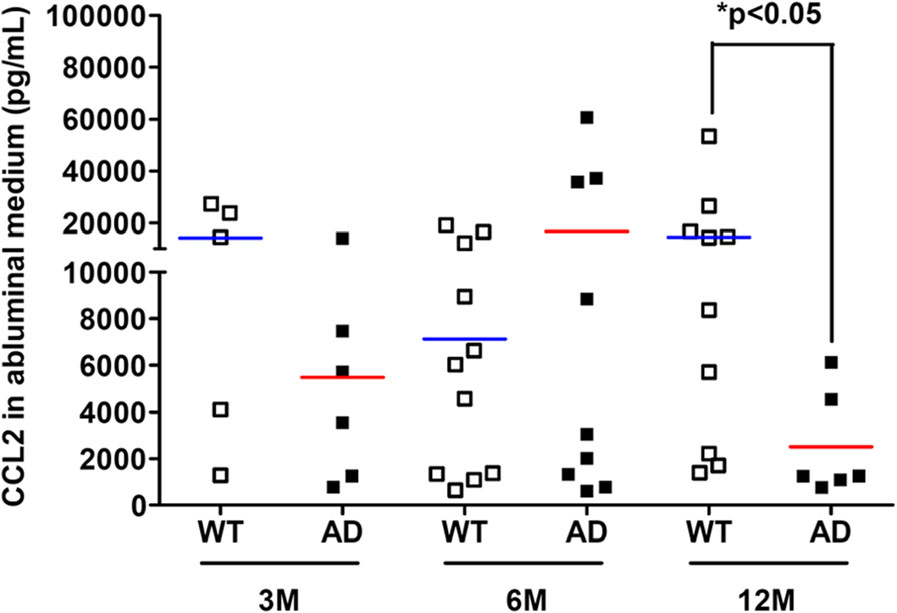
CCL2 expression in abluminal media (M2) from WT and AD BBB models at the ages of 3 months (*n* = 5 for WT, *n* = 6 for AD), 6 months (*n* = 11 for WT, *n* = 9 for AD), and 12 months (*n* = 10 for WT, *n* = 6 for AD). CCL2 levels were analyzed by the 5-plex Luminex® xMAP® assay (for CCL2, CCL3, CCL4, CCL5, and CXCL10) containing a mixture of beads specific for each chemokine as described in the “Methods” section. Chemokine levels are expressed in picograms per millilitre. The mean is represented by a colored line following the origin of cell types (blue for WT and red for AD cells, respectively). ^*^P < 0.05 in M2 of AD BBB models compared to WT BBB models at 12 months by Mann-Whitney’s test

Figure 5 shows a significant decrease of CCL2 levels by 5.78-fold in the abluminal media (M2) of AD BBB models compared to WT BBB models at 12 months. However, no difference was found in the CCL2 expression in the luminal media, PBMCs, and abluminal and luminal lysates between WT and AD BBB models whatever the age of mice (Table 2).

#### CCL3 levels

**Fig. 6:**
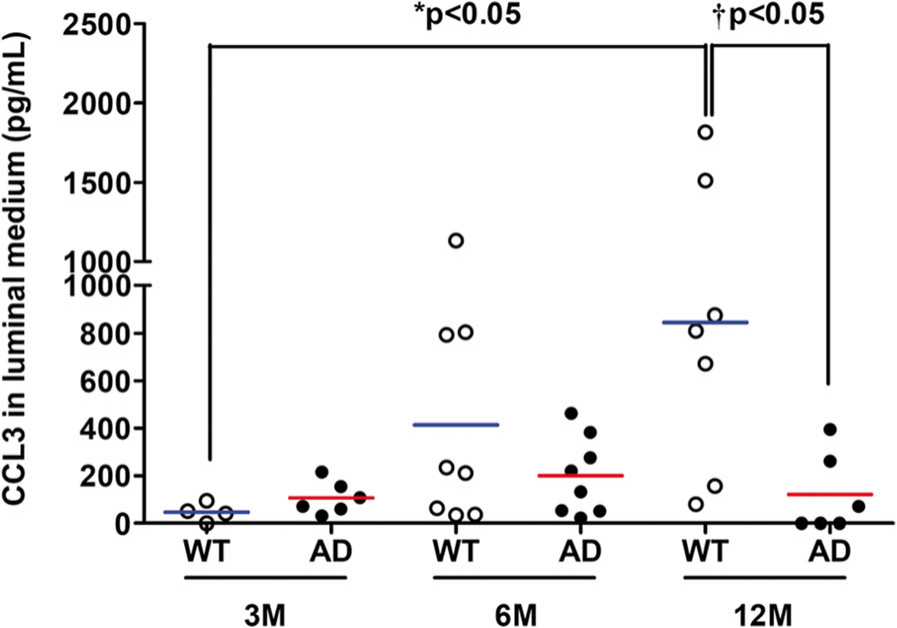
CCL3 expression in luminal media (M1) from WT and AD BBB models at the ages of 3 months (*n* = 4 for WT, *n* = 6 for AD), 6 months (*n* = 8 for WT and AD), and 12 months (*n* = 7 for WT, *n* = 6 for AD). CCL3 levels were analyzed by the 5-plex Luminex® xMAP® assay (for CCL2, CCL3, CCL4, CCL5, and CXCL10) containing a mixture of beads specific for each chemokine, as described in the “Methods” section. Chemokine levels are expressed in picograms per milliliter. The mean is represented by a colored line following the origin of cell types (blue for WT and red for AD cells, respectively). ^*^P < 0.05 in M1 from WT BBB models at 12 months compared to M1 from WT BBB models at 3 months by Kruskal-Wallis test with a Dunn’s multiple comparison test. ^†^*P* < 0.05 in M1 from AD BBB model compared to M1 from WT BBB models at 12 months by Mann-Whitney’s test

Results showed a significant increase by 18.19-fold of CCL3 expression in the luminal media (M1) of 12-month-old WT BBB models in comparison with 3-month-old WT BBB models. Moreover, this increase was also significant (by 6.97 fold) compared to 12-month-old AD BBB model (Fig. 6). However, in our experimental conditions, no difference was found in the CCL3 expression in the abluminal media, in PBMCs, and in luminal or abluminal lysates (Table 2).

## Discussion

Besides traditional hallmarks observed in AD, which are amyloid plaques and intraneuronal neurofibrillary tangles, neuroinflammation is now recognized as a prominent feature in AD [59]. Its deleterious or beneficial role during AD remains to be clarified, and it is still an object of debate in the scientific community. Interestingly, neuroinflammation in AD is not only caused by a central inflammation, but it is also subjected to a peripheral inflammation [10]. Indeed, Togo et al. [21] observed that T cells were present in postmortem brains of AD patients. The same was observed with the work of Simard et al. [60] where they showed that circulating monocytes were able to cross the BBB after irradiation of APP/PS1 mice, followed by the transplantation of GFP-expressing bone marrow cells. Interestingly, those GFP-expressing cells were found next to amyloid plaques. Still, we need to be cautious because irradiation can cause a loss in the integrity of the BBB, explaining alone why these cells were able to migrate through the BBB. Other authors demonstrated that after Aβ immunization of AAP/INF-γ mice, T cells with both subtypes CD4 and CD8 T cells were found at sites with amyloid plaques [61]. These results showed that peripheral cells are capable of crossing the BBB under specific conditions, to reach their targets. The fact that peripheral immune cells can contribute to neuroinflammation in AD is valuable, indeed some authors observed that T cells could be more efficient to eliminate amyloid plaques [62], with an autophagic activity that remained functional [49] in comparison with senescent microglial cells [63].

There is a need to have a better understanding of the mechanisms behind the migration of peripheral immune cells to the brain and how the chemotactic environment can evolve with aging or neuropathological conditions. Most of the data available come from studies of acute conditions like in mesenteric ischemia [64], or in viral [65] and bacterial [66] infections. Insights into changes of chemotactic environment during chronic neurodegeneration like AD are lacking. Therefore, our laboratory decided to conduct this work; we wanted to assess if there was a change of the chemotactic environment in an integrated mouse BBB model according to age and/or with AD pathology. How peripheral immune cells (primary WT or AD PBMCs) could impact, in terms of chemokine production, a modeled healthy brain parenchyma (or abluminal side) at the age of 3, 6, and 12 months in a WT or AD BBB model?

In our experimental conditions, no change was observed in terms of tightness and functionality of P-gp with aging in both WT and AD BBB models. At 3, 6, and 12 months, no modification of these parameters was observed between WT and AD BBB models. These results were consistent with other studies, where BBB dysfunction occurred also with aging [67], making it difficult to discriminate BBB dysfunctions between aging and AD. In addition, a recent study indicates that the expression profiles of studied genes (18 drug transporters and 4 tight junction-associated proteins) were similar in the brain tissues of 12–16-month-old AD (APPswePS1dE9, Tg2576, and APP/PS1 transgenic mice) and control mice. In the microvessel fraction in APPswePS1dE9 mice, > 2-fold alterations were detected in the expressions of 11 genes but none at the protein level [68]. Other authors found that BBB permeability was vastly spared in various mouse models of AD, including PS2-APP, Tau transgenics, and APOE4 knockin mice at 5–6 months and 15–16 months [69]. Recently, data indicated that the proportion of monocyte-derived macrophages (MDM) was increased in the brain of aged mice and suggested that MDM infiltration was a late process during the Aβ pathology as it was observed in 21-month-old APPswePS1dE9 mice [42].

### CX3CL1

The CX3C chemokine ligand 1, also known as CX3CL1 or “fractalkine,” is a member of the CX3C family. CX3CL1 exists as either a soluble form or as a membrane-anchored form. The membrane-bound form of CX3CL1 is made possible by its mucin stalk, which acts like an adhesion molecule, in particular with microglial cells during inflammation. This mucin stalk can be cleaved by the action of enzymes like ADAM-10 or ADAM-7 to produce the soluble form. Then, sCX3CL1 can act as a molecular signal and interacts with microglial cells which express the CX3CL1 receptor: CX3CR1 [70]. CX3CL1 is known to play a critical role in a crosstalk between neurons and microglial cells [71]. This chemokine is expressed in both a constitutive and inducible manner, in neurons, and especially in neurons found in the hippocampus and the cortex [70]. While neurons are one of the prominent sources of CX3CL1, its expression can be found in epithelial intestinal cells, in inflamed endothelium, and also in pericytes during AD [72–74]. Here in mouse BBB models, results showed that CX3CL1 expression is increased in the abluminal media (M2) at the age of 12 months only in AD BBB while its levels decreased in luminal lysates at 12 months and also in PBMCs at 3 months compared to WT PBMCs. Interestingly, an increase of its soluble form expression in the plasma has been found in patients with mild to moderate AD, while it was lower with patients with severe AD [75]. In the same way, other authors demonstrated that there is an increased expression of the CX3CL1 coding gene in the brain of patients with AD compared to healthy controls, especially in brain areas with a marked vulnerability in AD-related changes as the hippocampus. They also proposed CX3CL1 as a marker for early diagnosis of AD [76]. In addition, CX3CL1 increased in the brains of APPswePS1dE9 mice from the age of 10 months [42]. The positive or deleterious role of CX3CL1 in AD is still debated in the scientific community. Indeed, some authors found that using different isoforms of CX3CL1 in a gene therapy was effective to reduce neuron loss in a rat Parkinson’s disease model, suggesting a positive role of soluble CX3CL1 in neuroprotection [77]. In the same direction, Mizuno et al. [78] showed that CX3CL1 reduced oxide nitric level in a dose-dependent manner, involved in neuroinflammation, whereas some authors found that the absence of expression of CX3CR1 in three different mouse AD models helped to protect against neuronal death by preventing microglial activation; they also found a lower amount of amyloid plaques [41, 79]. CX3CL1 in this study through its interaction with microglial cells seemed to be deleterious in the pathogenesis of AD, and not neuroprotective. It is important to note that these results were obtained with young mice; indeed, mice were sacrificed at the age of 4 months in both transgenic mouse models. Other authors showed that CX3CL1 can have different effects over time, with a deleterious effect in the acute post-injury phase of a mouse model traumatic brain injury (TBI). On the contrary, they found a positive and neuroprotective impact of CX3CL1 in the chronic phase post-TBI. These different effects were related to changes in microglia phenotypes, with enhancing of the neuroprotective and anti-inflammatory state [80].

Results showed that CX3CL1 expression increased in the abluminal medium but decreased in the luminal lysates between 3M and 12M in our AD BBB models while it remained stable and very low in the PBMCs from AD mice. One may propose that this increased expression in M2 could be mainly explained by a release of CX3CL1 by the endothelial cells and pericytes present in the luminal compartment. Then, the soluble CX3CL1 would go to abluminal medium through AD BBB (pore of the insert is 0.4 μM).

Only a few authors have described a production of CX3CL1 by PBMCs; McComb et al. [81] found that mononuclear phagocytes can increase both CX3CR1 and CX3CL1 gene expression under a smoke exposure, and this CX3CL1-CX3CR1 pathway can promote a cell-survival signal [82], while it also allowed macrophages to amplify their signals and stimulated their migration. We also found a production of CX3CL1 in PBMCs from AD patients cultured alone, when treated with phytohemagglutinin [47].

### CCL2

CC chemokine ligand 2 (CCL2) is a member of the “Monocyte Chemoattractant Proteins” or MCPs family and is also named MCP-1. CCL2 exerts its biological effects through its receptor: CCR2. A wide number of cells are able to produce CCL2; indeed, CCL2 is expressed by smooth muscular cells, fibroblasts, epithelial cells, monocytes, astrocytes, microglial cells, and neurons [83].

We found that expression of CCL2 is decreased in the abluminal media (M2) at the age of 12 months in AD BBB model compared to WT BBB model at the same age. Martin et al. [42] showed no modification of CCL2 expression in the brain of APPswePS1dE9. Furthermore, it is known that serum CCL2 levels are increased in MCI and mild AD patients, while its level in severe AD is lower [84]. The same pattern can be observed in intrathecal levels of CCL2 except for severe AD where CCL2 levels stay elevated [85, 86]. Interestingly, this increased expression of CCL2 is also found in serum samples from normal elderly subjects [87]. Indeed, some authors showed that some cell types like vascular smooth muscular cells enhanced their CCL2 expression with aging, creating a sort of proinflammatory phenotype, leading both to chronic inflammation and arterial aging, and could enhance atherosclerosis [88, 89]. The role of CCL2 in AD is still highly controversial in the scientific community. Some authors showed that CCL2 deficiency drove a stronger and accelerated β-amyloidosis, a microglial dysfunction, and led to altered cognitive functions in a murine APP/PS1/CCL2 KO model [90]. On the contrary, over-expression of CCL2 in a murine APP/CCL2 model or CCR2 deficiency in several mouse AD models led to an accelerated β-amyloidosis, astrogliosis, microgliosis, and cognitive dysfunctions [91, 92]. It is hypothezised that the “balance” between the CCL2/CCR2 axis is critical, explaining why over-expression of CCL2 could lead to detrimental effects. A protective effect in early stages of AD has been described, while CCL2 could be neurotoxic later in the pathogenesis of AD [18]. It is important that in our experimental conditions, the AD luminal compartment but not the WT luminal compartment led to a decrease of CCL2 in a healthy brain parenchyma at 12 months.

### CCL3

CC-chemokine ligand 3 (CCL3) is a part of the C-C family; this chemokine is also known as macrophage inflammatory protein-1 (MIP1-α). CCL3 exerts its effects through the CCR5 receptor. We found that CCL3 expression is increased between the age of 3 and 12 months in the luminal media (M1) of WT BBB model. Interestingly, CCL3 levels in brain of WT mice slightly increased with age (significant results at 21 months compared to 6 months) and greatly increased in APPswePS1dE9 from the age of 6 months [42]. No difference in the literature has been described in the expression of CCL3 in CSF or serum samples between AD patients and healthy controls [93, 94]. The implication of CCL3 in the pathogenesis of AD seems both positive and detrimental: CCR5 KO murine models showed memory dysfunctions, with astrogliosis and amyloid plaques [39], whereas an over-expression of CCL3 led to synaptic process and memory dysfunctions [95]. Other authors showed that peripheral T cells can express CCL3 to enhance their transmigration through the BBB in AD [96].

We also studied the expression of three other chemokines: CCL4, CCL5, and CXCL10. While no change was observed in our experimental conditions, significant CCL4 levels increased in the brains of APPswePS1dE9 from the age of 6 months [42]. Furthermore, it is known elevated expression of CCL5 in the cerebral microcirculation of AD patients and that treatment of neurons with CCL5 results in an increase in cell survival and a neuroprotective effect against the toxicity of thrombin and sodium nitroprusside [97]. Alterations in blood CCL5 expression were evident at 3- and 6-month-old 3xTg-AD mice compared to WT animals [98]. Moreover, CXCL10 was found to be expressed in astrocytes in AD and detected in close proximity to Aβ plaques in a APPswe mouse model [99, 100]. CXCR3 (receptor of CXCL10) promotes plaque formation and behavioral deficits in APPswePS1dE9 mice [101].

## Conclusions

To conclude, these results highlight how a healthy modeled brain parenchyma in mouse BBB models can differently react in terms of chemokine production with aging or AD pathology. Indeed, we found that expression of abluminal CX3CL1 increased while abluminal CCL2 decreased in AD BBB models at the age of 12 months compared to WT BBB models. In contrast, CCL3 changes concerned the luminal compartment with an increase with aging while no modification was observed in AD BBB models whatever the age of mice. While CCL2 and CX3CL1 have a dual role in AD, results indicate that a healthy brain parenchyma can be controlled by luminal compartment with AD phenotype, suggesting the impact of this compartment in the AD pathophysiology. However, healthy luminal compartment controlled CCL3 levels at the BBB. In mouse, CCL4, CCL5, and CXCL10 did not influence by the phenotype of luminal compartment.

## Additional file

Additional file 1: Shematic representation of the BBB model. (PDF 129 kb)

## Abbreviations

AD: Alzheimer’s disease
Aβ: Beta amyloid
BBB: Blood-brain barrier
CNS: Central nervous system
CSF: Cerebrospinal fluid
DMSO: Dimethylsulfoxide
FD4: Fluorescein isothiocyanate-dextran 4kDa
IFN-γ: Interferon-gamma
IL: Interleukin
LPS: Lipopolysaccharide
MCI: Mild cognitive impairment
NaF: Sodium fluoride
NVU: Neurovascular unit
PBMCs/PB: Peripheral blood mononuclear cells
P-gp: P-glycoprotein
PHA: Phytohaemagglutin
PMSF: Phenylmethylsulfonyl fluoride
PS: Penicillin/streptomycin
TEER: Trans-endothelial electric resistance
WT: Wild type
