# Supplementary material for: Longitudinal chemokine profile expression in a blood-brain barrier model from Alzheimer transgenic versus wild-type mice
Source: J Neuroinflammation. 2018 Jun 13;15:182. doi: 10.1186/s12974-018-1220-7 (PMC6001165; doi:10.1186/s12974-018-1220-7)
Supplement: Supplementary file 1 — Shematic representation of the BBB model. (PDF 129 kb) [file 12974_2018_1220_MOESM1_ESM.pdf]

Additional File 1

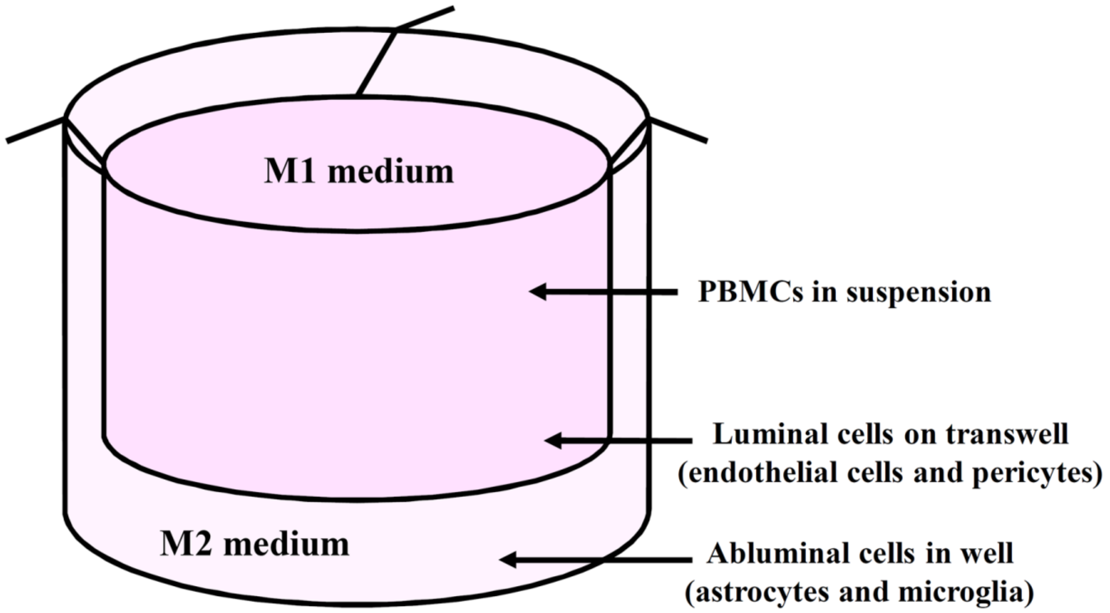

Additional file 1: Shematic representation of the BBB model.

As stated in the Material and Methods, the luminal part is either of AD or WT phenotype and the abluminal part is always WT. All the cells that make up this model are primary cultures.
